# Transmission and Surveillance of Rat Hepatitis E Virus in Swine

**DOI:** 10.3201/eid3008.240484

**Published:** 2024-08

**Authors:** Matheus Filgueira Bezerra, Maria Gabriela Oliveira da Paz, Edmilson Ferreira de Oliveira-Filho, Christian Robson de Souza Reis

**Affiliations:** Oswaldo Cruz Foundation, Recife, Pernambuco, Brazil (M.F. Bezerra, M.G.O. da Paz, C.R.D.S. Reis);; Humboldt-Universität zu Berlin, Berlin, Germany (E.F. de Oliveira-Filho).

**Keywords:** Rat HEV, Hepatitis E Virus, viruses, swine, one health, rodentborne, zoonoses

**To the Editor:** The study by Rios-Muñoz et al. reporting rat hepatitis E virus (HEV) RNA in swine feces contains intriguing findings with the potential to change our understanding of rat HEV transmission routes (*1*). One relevant aspect highlighted by the authors is that limited prior evidence of rat HEV infection in swine might be partially explained by the lack of rat HEV serology tests for pigs. The low genomic homology between rat HEV and other HEV (<60%) makes most of the commercially available HEV-based tests ineffective in detecting rat HEV (*2*,*3*).

Such findings are exciting but must be interpreted with caution because some gaps remain to be addressed. Pigs can feed on small mammal remains and even prey on rodents, which means detecting rat HEV RNA in pig feces does not conclusively indicate an infection. To rule out the possibility of viral detection because of contaminated food, it is necessary to detect the virus in other tissues, such as blood or liver (*3*). Of note, a substantial proportion of the positive samples in the study by Rios-Muñoz et al. exhibited high cycle threshold values, which might be suggestive of residual viral RNA. Furthermore, considering the hypothesis that both viruses could be transmitted through contaminated swine meat, it remains unclear why rat HEV infection in humans is uncommon when compared with other HEV.

Nevertheless, from our perspective, these findings suggest the possibility of approaching swine as a sentinel species. If results are confirmed in additional eco-epidemiologic studies, sampling swine stools could offer valuable public health information. Insights from other rodentborne diseases, such as bubonic plague, underscore the benefits of a surveillance strategy focused on sentinel species rather than primary hosts (*4*). Sampling rodents is logistically more challenging and expensive than for domestic animals, but surveillance of sentinel species is typically more efficient in predicting the dissemination of zoonotic diseases at early stages.
